# Cash transfers and HIV/HSV-2 prevalence: A replication of a cluster randomized trial in Malawi

**DOI:** 10.1371/journal.pone.0210405

**Published:** 2019-01-31

**Authors:** Lynette M. Smith, Nicholas A. Hein, Danstan Bagenda

**Affiliations:** 1 Department of Biostatistics, University of Nebraska Medical Center, Omaha, Nebraska, United States of America; 2 Department of Anesthesiology, University of Nebraska Medical Center, Omaha, Nebraska, United States of America; INSERM Unité 897, FRANCE

## Abstract

**Introduction:**

In this paper we perform a replication analysis of “*Effect of a cash transfer programme for schooling on prevalence of HIV and herpes simplex type 2 in Malawi*: *a cluster randomised trial”* by Sarah Baird and others published in “*The Lancet”* in 2012. The original study was a two-year cluster randomized intervention trial of never married girls aged 13–22 in Malawi. Enumeration areas were randomized to either an intervention involving cash transfer (conditional or unconditional of school enrollment) or control. The study included 1708 Malawian girls, who were enrolled at baseline and had biological testing for HIV and herpes simplex virus type 2 (HSV-2) at 18 months. The original findings showed that in the cohort of girls enrolled in school at baseline, the intervention had an effect on school enrollment, sexual outcomes, and HIV and HSV-2 prevalence. However, in the baseline school dropout cohort, the original study showed no intervention effect on HIV and HSV-2 prevalence.

**Methods:**

We performed a replication of the study to investigate the consistency and robustness of key results reported. A pre-specified replication plan was approved and published online. Cleaned data was obtained from the original authors. A pure replication was conducted by reading the methods section and reproducing the results and tables found in the original paper. Robustness of the results were examined with alternative analysis methods in a measurement and estimation analysis (MEA) approach. A theory of change analysis was performed testing a causal pathway, the effect of intervention on HIV awareness, and whether the intervention effect depended on the wealth of the individual.

**Results:**

The pure replication found that other than a few minor discrepancies, the original study was well replicated. However, the randomization and sampling weights could not be verified due to the lack of access to raw data and a detailed sample selection plan. Therefore, we are unable to determine how sampling influenced the results, which could be highly dependent on the sample. In MEA it was found that the intervention effect on HIV prevalence in the baseline schoolgirls cohort was somewhat sensitive to model choice, with a non-significant intervention effect for HIV depending on the statistical model used. The intervention effect on HSV-2 prevalence was more robust in terms of statistical significance, however, the odds ratios and confidence intervals differed from the original result by more than 10%. A theory of change analysis showed no effect of intervention on HIV awareness. In a causal pathway analysis, several variables were partial mediators, or potential mediators, indicating that the intervention could be working through its effect on school enrollment or selected sexual behaviors.

**Conclusions:**

The effect of intervention on HIV prevalence in the baseline schoolgirls was sensitive to the model choice; however, HSV-2 prevalence results were confirmed. We recommend that the results from the original published analysis indicating the impact of cash transfers on HIV prevalence be treated with caution.

## Introduction

Young women between the ages of 15 and 24 represent approximately 30 percent of new human immunodeficiency virus *(*HIV) infections in southern Africa, compared to only 6 percent of young men in the same age group[1]. Prevention strategies have focused on this high risk population in an attempt to have the greatest impact in controlling the HIV epidemic. One of the potential causes of the age and gender disparities in HIV infections is the high frequency of relationships between younger women with older men in southern Africa[1]. These relationships may be motivated by financial necessity on the part of young women, hence there has been a focus on the use of cash transfers as a potential prevention strategy. If young women turn to relationships with older men due to financial need, cash transfers could mitigate that need.

Cluver and others found that receipt of a cash transfer was associated with reduced incidence and prevalence of transactional sex and age-disparate sex in girls aged 12–17, in an observational study conducted in South Africa[2]. Hallfors and colleagues conducted a three-year cluster randomized control trial where the intervention consisted of subsidized school costs for orphan adolescent girls[3]. While other beneficial effects were found, such as improved likelihood to stay in school, socioeconomic status and reduced likelihood to marry in the intervention group versus controls, there was no difference in HIV or HSV-2 prevalence after five years[3]. Pettifor *et*.*al*. completed a randomized clinical trial of young women in rural South Africa, looking at the effect of conditional cash transfers on HIV incidence[4]. They found that HIV incidence did not differ between girls who received the cash transfer and those who did not. However, they did find that school attendance significantly reduced the risk of HIV infection, regardless of whether the girl was in the intervention or control group. Other studies have shown associations of improved economic empowerment of young women through microfinance loans or subsidies to pay for school uniforms or other education costs, but typically have only measured sexual behavior post-intervention or measured other sexually transmitted infections as a proxy for sexually risky behavior, rather than HIV prevalence directly[5–7].

One of the first, most highly impactful studies in HIV research on cash transfers in young women, is *Effect of a cash transfer programme for schooling on prevalence of HIV and herpes simplex type 2 in Malawi*: *a cluster randomised trial*, by Baird *et*.*al*., published in *The Lancet* in 2012[8]. This study uses a fairly new approach to address structural drivers of HIV/AIDS described as physical, social, cultural, organizational, community, economic, legal or policy aspects of the environment that influence the risks and vulnerability environment and thus act as barriers to, or facilitators of, HIV prevention and treatment behavior[9,10]. The impact of this study lies both in the study population considered and the absence of intentional HIV prevention training during the intervention. The authors found that monthly cash transfers, which were not accompanied by a program or training directly related to HIV prevention, were associated with decreases in the prevalence of both HIV and herpes simplex virus 2 (HSV-2) at 18 months, as well as decreases in high-risk sexual behavior of the cohort of baseline schoolgirls. These original analysis results suggest that the structural intervention of cash transfer alone was enough to affect behavior. Specifically, they suggest that baseline schoolgirls in the intervention group were more likely to choose younger partners and report less frequent sex with those partners, even though the study found no effect on the frequency of unprotected sex. In the baseline school dropout cohort, they observe that the intervention group was also found to be more likely to report less frequent sex compared to the control group. However, this positive effect of the cash transfers on decreasing the HIV and HSV-2 prevalence is in contrast to several other studies discussed above, notably the more recent study by Pettifor *et*.*al*. that utilizes a simpler designs and measures HIV incidence rather than prevalence[11].

In this paper, we performed a replication analysis of the study conducted by Baird and colleagues[8]. As part of the replication process, we developed a replication plan which was finalized and approved prior to conducting any analysis. The full replication plan is published online as well as the full replication report[12,13]. We selected this study for replication due to its high impact in the field of HIV and HSV-2 prevention and its complex study design that lends itself to multiple analysis methods that could potentially impact the findings that were observed. The study by Baird *et*. *al*. employed a complex cluster randomized design which included two separate cohorts, a weighting strategy of the observations based on the probability of selection, as well as two stratification factors. Data collected from this type of design can be analyzed by a number of valid methods. The background and necessity of replication, as well as our selected alternate analytical strategies, namely, measurement and estimation analysis (MEA), and theory of change analyses are described in detail by Brown and others[14].

The replication included three objectives: perform a pure replication, a measurement and estimation analysis, and a theory of change analysis. The pure replication attempts to reproduce the results presented in the paper using the author’s cleaned data set and reported statistical methods from the original paper. In the MEA section of the report, we explored some alternate analysis strategies to determine the robustness of the results, focusing on outcomes that were statistically significant or close to being statistically significant in the original paper. To extend the study, three different theory of change analyses were considered: 1) using principal component analysis, a composite HIV awareness variable was created that can be used to examine the effects of the treatment on HIV awareness; 2) wealth indices were constructed to determine if the cash transfer intervention would be more effective in poorer households; and 3) a causal pathway was explored to determine the direct effect of being enrolled in school and risky sexual behaviors on HIV and HSV-2 prevalence at 18 months.

## Methods

### Background

The original study was conducted in the Zomba district of southern Malawi, which is made up of 550 enumeration areas (EAs) and tends to have high poverty rates, high HIV prevalence and low school enrollment[8]. Never married girls who were aged 13−22 years old were eligible for the study. A cluster randomized trial was used to assess the effect of a cash transfer intervention on primary outcomes: HIV, HSV-2 prevalence, and school enrollment; and secondary outcomes: syphilis prevalence, HIV knowledge, and risky sexual behaviors. 176 EAs were selected out of a total of 550 from three geographic strata: urban, near rural, and far rural. Biological outcomes (HIV, HSV-2, and syphilis prevalence) were collected in 104 selected EAs (52 intervention and 52 control) at 18 months follow-up.

Selected EAs were randomized to intervention (cash transfer) or control (no cash transfer), stratified by geographic location. Two cohorts were defined: those enrolled in school at baseline (baseline schoolgirls) and those not enrolled in school at baseline (baseline dropouts). Percentage of baseline schoolgirls selected varied by age group and geographic location, but all baseline dropouts were selected[8]. For the baseline schoolgirls cohort, the EAs assigned to the intervention group were further randomized to a conditional cash transfer (CCT) group, where the girl was required to attend school to receive payment, or an unconditional cash transfer (UCT) group, where no school attendance was required. The baseline dropout cohort intervention group was assigned to CCT. Written informed consent was obtained from participants and guardians of girls younger than 18 years old. The original study design was approved by the ethics review committees at the University of California at San Diego (USA) and the National Health Sciences Research Council (Malawi).

### Data

The database for the replication analysis was downloaded from the World Bank website on January 18, 2016, and included the round 1 baseline data and the round 2 outcome data[15]. The original study includes three data sets: baseline, follow-up and test results for HIV, HSV-2 and syphilis. The original authors pooled the relevant survey questions from baseline, follow-up and the test results into one deidentified merged public data set. User guides for the baseline and follow-up data sets and Stata code used to analyze the data for the original paper were obtained from the World Bank website. For our analysis, we used the deidentified merged public data set; which ensures that the same sampling was used as employed by the original authors, but at the cost of independently constructing the sampling weights.

### Statistical methods

#### Pure replication methods

The original paper conducted analyses for each cohort separately. Unadjusted and adjusted ORs were computed using logistic regression models, with robust standard errors, which allows for intraclass correlation. Sampling weights were utilized to account for probability of inclusion. Adjusted models included age group, geographical stratum, and baseline level of the outcome variable when available. Heterogeneity of intervention effects was assessed for CCT and UCT groups in the baseline schoolgirls cohort. The analysis was performed in Stata version 10.1 (Stata Statistical Software. College Station, TX: StataCorp LLC). It is unclear if the original statistical analysis plan was pre-specified. Therefore, we cannot determine which of the analysis decisions may have been made post-hoc, such as the type of statistical model used. In addition, we did not have access to the detailed sample selection plan and thus the sampling weights utilized in the analysis could not be verified independently.

For the replication analysis, the data was converted from Stata to SAS using Stata software version 14.1. The replication analysis was conducted using the same methods as the original analysis, using SAS/STAT software version 9.4 (SAS Institute Inc., Cary, North Carolina, USA) and Stata version 14.1. The SAS SURVEY procedures were used for this analysis. The survey procedures include domain statements that allow for subgroup analysis, weight statements for weighting of observations according to the study design and clustering for the primary sampling unit (EA) and stratum. This methodology allows for the computation of unadjusted and adjusted OR, with robust standard errors and the inclusion of sampling weights. Differences in the confidence intervals (CIs) occurred depending on whether the analysis accounted for stratum and subpopulation analysis/domain analysis. Most of these are in the hundredths decimal place, are not considered to be discrepancies and do not change the results of the paper. If there was a difference in the sample size or in the point estimate, we did recognize this as a discrepancy.

#### MEA methods

Due to the design of this study which has multiple levels of complexity, including cluster randomization, unequal sampling strategy which requires a weighted analysis, further randomization into UCT and CCT among the intervention group, subsampling of clusters for the biological endpoints, there is no “preferred” analysis method. Therefore, in the MEA portion of the replication, we compare alternative estimation strategies that are valid for the analysis of this type of complex data, which we pre-specified in an approved analysis plan prior to obtaining the data.

The MEA robustness checks focused on primary and secondary outcomes from the original paper. The baseline schoolgirls and baseline dropouts are treated as two separate cohorts for analysis, as in the original paper.

Generalized linear mixed models (GLMMs), can be used for the analysis of cluster randomized trials as described by Rabe-Hesketh and Skrondal and Pfefferman, which include both random and fixed effects[16,17]. Baseline measurements for behavior outcomes, age and stratum were included as fixed effects, and EA as a random effect to account the clustered nature of the data. Scaled weights were included to avoid bias[17]. We pre-specified that if the GLMM estimated ORs differed from those initially reported by more than 10 percent for the primary outcome variables (HIV and HSV-2), it was concluded that the results are somewhat sensitive to the model choice.

Generalized estimating equations (GEE) methodology was used as another robustness check[18]. The GEE models included the intervention effect and the same adjustment variables as the GLMM model, as well as sampling weights, and an exchangeable correlation structure for the EA clusters. GEE is similar to the method used by the original authors and is robust to misspecification only if the mean is correctly modeled. Both are known as robust standard errors methods and utilize sandwich estimators in the estimation step.

Group permutation-based methods that account for the cluster randomization are used to explore the critique by Webb that the results are sensitive to the adjustment of weights and cluster size[19]. In the permutation testing, the EA is considered to be the experimental unit, and thus accounts for the intraclass correlation within EAs by permuting the areas rather than individuals[20]. The permutation test statistic used is the difference in overall average between the control and experimental groups. These methods are useful when asymptotic theory does not hold since they require few distributional or modeling assumptions. Permutation methods are described in more detail in the online report[13].

Cluster-level statistics were calculated from the GLMM analysis, as recommended by the CONSORT guidelines[21]. Intraclass correlation (ICC) was calculated using the ANOVA estimator along with 95% CIs using a modified Wald test.

#### Theory of change methods

In our pre-specified replication plan, three different theory of change analyses were considered to extend the initial analysis. A composite HIV awareness variable was created using principal component analysis (PCA), based on several of the survey variables, including whether the study participant: has had an HIV test, knows a healthy-looking person can have HIV, knows that HIV can be transmitted through breastfeeding and received health training about HIV/AIDS. The intervention effect on the composite HIV variable was examined using a linear regression model with PROC SURVEYREG, adjusting for baseline levels of knowledge, age, and stratum. An interaction between age and HIV awareness was considered.

PCA was also used to create two wealth indices using variables collected at baseline. A family wealth index included variables: mother alive, father alive, female-headed household. The family wealth index had positive weighting for mother alive and father alive and negative weighting for female-headed household. Higher levels of family wealth index indicates that the girl has more familial support at home. An item wealth index included: household owns a television, has access to a mobile telephone, electricity and piped water available[22]. All the item wealth index variables had positive weights, with higher levels indicating more material wealth at the girl’s home. The wealth index variables were tested in a multiple logistic regression model, along with the intervention, their interaction, age group, and stratum with PROC SURVEYLOGISTIC.

**Fig 1:**
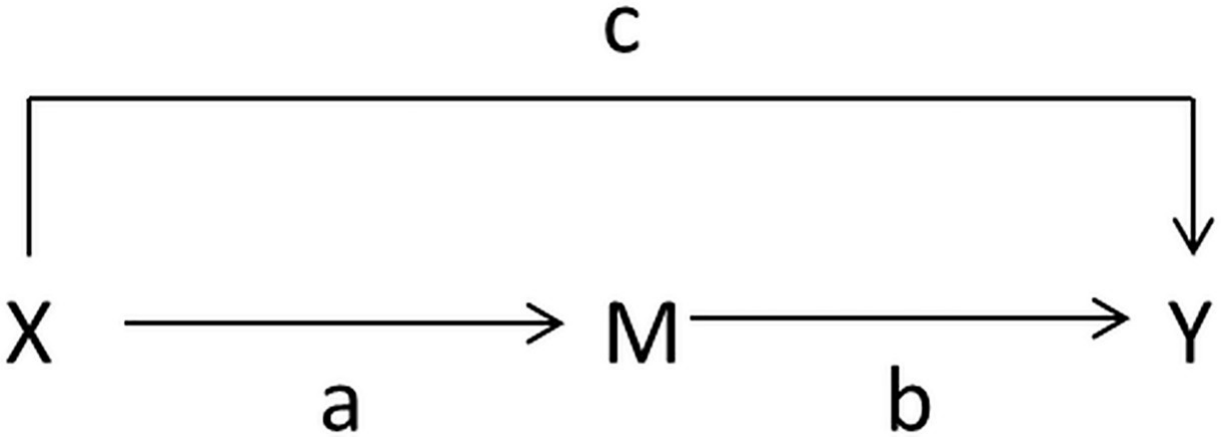
Mediator pathway. To test the mediator pathway the models in Fig 1 were run.

Pathway-specific effects were explicitly investigated to see how much of effect of the intervention were mediated through reduced sexual behavior and enrollment in school. To do this, a four-step approach proposed by Baron and Kenny was used[23]. This method involves a series of four regression models shown pictorially in Fig 1. X is the intervention variable; M is the mediator variable (school enrollment or risky sexual behaviors); Y is the outcome variable; a and b are direct effects; and c is the direct effect of X on Y.

If one or more of these relationships are not significant, then we can conclude that mediation is not likely. If relationships exist in tests 1−3, then step 4 is considered. If the effect for M in the multiple regression model is significant, then the conclusion is there is some form of mediation; if X is not significant, then it is full mediation; if both are significant, then the model supports partial mediation. These series of tests are shown in Table 1. The models included survey weights and clusters, adjusting for age group, stratum and baseline level using PROC SURVEYLOGISTIC.

**Table 1:** Statistical tests used to examine the mediator pathway.

	Analysis
Test 1	Predict Y with X to test for path c.
	E[Y] = B_0_+B_1_X
Test 2	Test for path a, the effect of X on M.
	E[M] = B_0_+B_1_X
Test 3	Test for path b, the effect of M on Y.
	E[Y] = B_0_+B_1_M
Test 4	Multiple regression with X and M predicting Y.
	E[Y] = B_0_+B_1_X+B_2_M

## Results

### Push button replication

As part of the replication study we conducted a push button replication (PBR) where we ran the data analysis code provided by the authors to reproduce the tables in the original paper. The PBR was found to be comparable or incomplete, meaning that the code supplied by the original authors did not produce all the results found in the paper. The code needed to be modified to reproduce all tables. The full PBR results can be found online[13].

### Pure replication

The pure replication reproduced the baseline and main outcome tables of the study. The results are summarized here and full results can be found online[13]. Our results were almost identical to the original paper; however, there were a few discrepancies. The numbers presented in the original tables showed 52 EAs in the control group and intervention groups and 25 in the CCT and 27 in the UCT groups. The pure replication results found the number of EAs in the CCT arm to be 26, not 25 as reported in the original paper. It was determined that one of the EAs appears in both CCT and UCT groups. Other sample size differences were noted, and we concluded sample sizes were taken from the marginal totals from one of the variables analyzed, which had a few missing data points (about 0.2%). One obvious typo was found, the original paper reported a sample size of 299 for the survey question “had sexual intercourse once per week” for the baseline school girls which should be 499. There were two point estimates of OR that did not match and could not be explained by rounding errors, corresponding to the outcomes of syphilis prevalence and had unprotected sexual intercourse. The discrepancies in these point estimates and confidence intervals (CI) did not affect the significance or interpretation of the results.

The original authors found that the intervention lowered the odds of HIV and HSV-2 prevalence in baseline schoolgirls, but did not have a significant effect for baseline dropouts. The pure replication matched these results within rounding error, except for upper confidence limit for HIV prevalence in the baseline dropout group, which we found to be 2.63 compared to 2.61. In the analysis of the heterogeneity of the treatment arms in the baseline schoolgirls, we found the CI for the HIV prevalence in the CCT versus control changed enough that the borderline significant difference shown in the original analysis is now non-significant by a small margin. In our opinion, we do not think these differences change the original authors’ findings.

### MEA

The MEA explores the robustness of the findings through using alternative, but valid forms of analysis. There is currently no gold standard analysis method for this type of data, and alternate methods can be utilized to determine if the results are dependent on the analysis method chosen. We explored three analysis methods–GEE, GLMMs, and permutation.

CONSORT guidelines for cluster randomized trials were examined and compared to what was reported in the original study. Some cluster level details are not explicitly reported in the original study, such as eligibility criteria for clusters, whether the objective/hypothesis or primary outcome pertains to the cluster, participant, or both. As part of our replication study, cluster-level summary statistics were calculated as recommended by the CONSORT statement. The number of individuals per cluster had a wide range, with 1 to 41 girls per cluster. The median number of baseline schoolgirls in the control clusters was quite different from the intervention group. The median number of baseline schoolgirls in the control group clusters was 15 (range: 3 to 41) compared to 7.5 in the intervention group (range: 1 to 37). In the baseline dropout cohort, the median number of girls per cluster was the same in the control clusters (median: 3, range: 1 to 14) and in the intervention clusters (median 3: range: 1 to 21). The ICC values tend to be very small, most near zero, with a median ICC of 0.03 (range: -0.03 to 0.24). Only the baseline dropout cohort had two ICCs that were somewhat higher near 0.2 (full results are reported online)[13]. The study flow diagram gives some insight to the reason for lower number of baseline schoolgirls in the intervention group clusters[8]. Enumeration areas were equally randomized to treatment and control, with similar numbers of girls allocated. The treatment group EAs were then further randomized to conditional and unconditional cash transfers. In order to test spillover effect of the intervention, 44% of the schoolgirls in intervention group were not offered cash and did not have testing for HIV and HSV-2. These girls were not included in the analysis of the original paper, leading to unequal number of schoolgirls in the clusters with HIV/HSV-2 test results. However, all the baseline dropout girls were offered cash transfers.

Table 2 provides the number and percentage in the intervention and control groups, adjusted ORs and 95% CIs from the original analysis, GEE, and GLMM. The GEE results are very similar to the original study results, whereas the GLMM odds ratio estimates and CIs are quite different. With the GLMM model, the intervention effect for baseline schoolgirls was not statistically significant after adjusting for baseline characteristics. The OR point estimate for HIV in the baseline schoolgirls increased by slightly more than 50% and is no longer statistically significant, whereas, HSV-2 OR changed by 40%, but they do not change statistical significance. In the GLMM analysis, the intervention was found to reduce the likelihood of sexual debut, compared to the control group, but the original analysis did not find a significant reduction. For HIV and HSV-2 prevalence in the baseline schoolgirls, the GEE results agree with the original results showing similar OR, CI, and a significant intervention effect after adjusting for age and stratum. In the baseline dropout cohort, the point estimate for OR and CIs differed between the three methods, but did not change statistical significance. Some of these differences in the models may be explained by the small proportion of events. The prevalence of HIV and HSV-2 is very small in the baseline schoolgirl’s cohort with only 1% in the intervention group and 3% in the control group having HIV and HSV-2 at 18 months. Many of the clusters have no HIV or HSV-2 positive girls at follow-up. For example, 38 out of 52 control EAs have no HIV positive girls and only 14/52 EAs have 1 or 2 positive girls. For the intervention EAs, 46 out of 52 EAs have no HIV positive girls and 6/52 have 1 or 3 positive girls.

**Table 2:** Effects of cash transfer intervention on outcome measures comparing original results to GEE and GLMM.

		Intervention	Control	Original	GEE		GLMM	
				Adjusted odds ratio	Adjusted odds ratio		Adjusted odds ratio	
	Outcome	n/N (%)^	n/N (%)^	(95% CI)	(95% CI)	P-Value	(95% CI)	P-Value
Schoolgirls								
	HIV prevalence	7/490 (1%)	17/799 (3%)	0.36 (0.14–0.91)	0.36 (0.14, 0.90)	0.029	0.54 (0.19, 1.54)*	0.250
	HSV-2 prevalence	5/488 (<1%)	27/796 (3%)	0.24 (0.09–0.65)	0.24 (0.09, 0.65)	0.005	0.34 (0.14, 0.83)	0.019
	Enrolled during 2008 school year	419/484 (90%)	669/801 (84%)	1.62 (1.07–2.45)	1.66 (1.09, 2.51)	0.017	1.29 (0.86, 1.92)	0.215
	Had sexual partner >25 years	4/**500** (<1%)	20/**826 (2%)**	0.21 (0.07–0.62)	0.21 (0.07, 0.62)	0.005	0.31 (0.11, 0.87)	0.026
	Had unprotected sexual intercourse	49/500 (8%)	63/826 (7%)	1.08 (0.67–1.75)	1.06 (0.66, 1.70)	0.818	1.07 (0.69, 1.67)	0.760
	Had sexual intercourse once per week	22/**499** (3%)	62/826 (7%)	0.46 (0.26–0.82)	0.45 (0.25, 0.81)	0.008	0.56 (0.33, 0.97)	0.038
	Sexual debut	39/371 (8%)	100/645 (13%)	0.64 (0.38–1.07)	0.65 (0.39, 1.10)	0.107	0.61 (0.40, 0.93)*	0.023
Dropouts								
	HIV prevalence	23/210 (10%)	17/207 (8%)	1.37 (0.72–2.61)	1.28 (0.69, 2.38)	0.440	1.44 (0.76, 2.74)	0.267
	HSV-2 prevalence	17/211 (8%)	17/208 (8%)	1.03 (0.47–2.24)	1.05 (0.48, 2.29)	0.908	1.08 (0.45, 2.55)	0.865
	Enrolled during 2008 school year	124/219 (57%)	27/220 (12%)	8.77 (5.07–15.1)	9.14 (5.36, 15.61)	< .0001	10.02 (5.40, 18.58)	< .0001
	Had sexual partner >25 years	20/225 (8%)	23/223 (10%)	0.79 (0.42–1.50)	0.76 (0.41, 1.43)	0.399	0.93 (0.48, 1.80)	0.820
	Had unprotected sexual intercourse	59/225 (25%)	64/222 (29%)	0.74 (0.44–1.23)	0.71 (0.43, 1.17)	0.173	0.76 (0.46, 1.26)	0.283
	Had sexual intercourse once per week	43/225 (19%)	66/223 (30%)	0.53 (0.32–0.86)	0.53 (0.32, 0.86)	0.011	0.53 (0.33, 0.85)	0.009
	Sexual debut	18/72 (26%)	27/72 (38%)	0.70 (0.33–1.45)	0.72 (0.36, 1.44)	0.352	0.67 (0.32, 1.37)	0.265

Note:

* Result changed from statistically significant to non-significant or non-significant to statistically significant when compared to the original paper.

^ The table displays the unweighted counts and weighted percentages.

Bold values are the replication study results and are slightly different from the original study due to typos.

The results of the original, GEE and GLMM analysis in the CCT and UCT groups compared to control in the baseline schoolgirls can be found in Table 3. GEE and original results were similar, but the GLMM model showed some differences. When modeling using GLMM, the adjusted ORs are not significant when comparing HIV prevalence in the CCT group versus the control group. HSV-2 results are also sensitive in the CCT group, changing by 59 percent, but they do not change statistical significance. Of the sexual behavior outcomes, had sexual partner ≥25 years changed to be non-significant in the CCT arm and sexual debut changed to significant in the UCT arm in the GLMM analysis.

**Table 3:** Effects of conditional or unconditional cash transfers on baseline schoolgirls by outcome measures, original compared to GEE and GLMM.

			Original			GEE			GLMM		
	CCT group	UCT group	CCT vs control (adjusted odds ratio [95% CI])	UCT vs control (adjusted odds ratio [95% CI])	p^	CCT vs control (adjusted odds ratio [95% CI])	UCT vs control (adjusted odds ratio [95% CI])	p^	CCT vs control (adjusted odds ratio [95% CI])	UCT vs control (adjusted odds ratio [95% CI])	p^
Enrolled during the 2008 school year	207/229 (92%)	212/255 (87%)	2.08 (1.14–3.82)	1.22 (0.77–1.96)	0.14	2.11 (1.14, 3.90)	1.24 (0.78, 1.98)	0.14	1.81 (1.13, 2.90)	1.01 (0.61, 1.67)	0.06
Sexual debut†	18/166 (7%)	21/205 (10%)	0.58 (0.29–1.15)	0.72 (0.37–1.40)	0.62	0.58 (0.29, 1.18)	0.74 (0.38, 1.45)	0.60	0.68 (0.38, 1.22)	0.56 (0.33, 0.96)*	0.60
Unprotected sexual intercourse	30/235 (9%)	19/265 (8%)	1.17 (0.67–2.05)	0.96 (0.50–1.83)	0.59	1.15 (0.66, 2.00)	0.93 (0.49, 1.76)	0.57	1.39 (0.84, 2.30)	0.79 (0.43, 1.45)	0.10
Had sexual intercourse once per week	14/235 (3%)	8/264 (3%)	0.53 (0.26–1.07)	0.37 (0.16–0.85)	0.49	0.52 (0.25, 1.07)	0.36 (0.16, 0.83)	0.49	0.76 (0.41, 1.42)	0.40 (0.18, 0.86)	0.16
Had a sexual partner aged ≥ 25 years‡	1/235 (<1%)	3/235 (1%)	0.08 (0.01–0.60)	0.36 (0.11–1.19)	0.19	0.08 (0.01, 0.62)	0.37 (0.11, 1.20)	0.20	0.17 (0.02, 1.14)*	0.42 (0.13, 1.37)	0.40
HIV prevalence‡	4/233 (1%)	1/255 (<1%)	0.29 (0.09–0.98)	0.47 (0.14–1.59)	0.57	0.30 (0.09, 0.98)	0.47 (0.14, 1.59)	0.57	0.42 (0.12, 1.51)*	0.65 (0.17, 2.40)	0.60
HSV-2 prevalence‡	1/235 (1%)	0/256 (0%)	0.37 (0.13–1.03)	0.08 (0.01–0.58)	0.16	0.37 (0.13, 1.03)	0.08 (0.01, 0.58)	0.16	0.59 (0.23, 1.50)	0.12 (0.02, 0.81)	0.12

^heterogeneity of odds ratio p-value

* Significance level is in a different direction from the original result.

†Cumulative risk measure, so no adjustment made for baseline status.

‡No adjustment for baseline measure because data not collected at baseline.

Permutation testing was also utilized, because it does not rely on distributional or modeling assumptions. When testing the effect of intervention on HIV prevalence in baseline schoolgirls,10,000 permutations resulted in a non-significant intervention effect in the unadjusted and adjusted models (p = 0.11 and p = 0.07, respectively). For HSV-2 the test gives a significant intervention effect in both unadjusted and adjusted models (p = 0.008 and p = 0.009, respectively). The effect of the cash transfer program on HIV prevalence was not statistically significant, but highly significant for HSV-2 prevalence by permutation test.

### Theory of change

The study was extended in a theory of change analysis in three ways: 1) by directly evaluating the effects of the intervention on improving the HIV awareness; 2) wealth indices for the participants was computed and then evaluated whether it influenced the effect of the intervention through an interaction; and 3) the causal pathway implied by the study was tested[24].

#### HIV awareness results

The intervention did not have a significant effect on HIV awareness for either baseline schoolgirls or baseline dropouts (p>0.05). Neither cohort showed a significant interaction between age and intervention in their effect on HIV awareness. The best predictor of HIV awareness at 12 months was baseline knowledge and in the baseline schoolgirls, being older was also a significant predictor of HIV awareness.

#### Wealth index results

One might expect that the cash transfer intervention would be most effective in poorer households. As Pettifor and others point out, “conditioning payments on school attendance may only be relevant in settings where there is a financial barrier to schooling”[11]. By looking for interactions with the wealth indices, we can determine if this type of intervention is unequally effective based on the wealth of the individual. The baseline dropout group may be in most need of the cash transfers to attend school and most at risk. With the wealth indexes, it can be determined if the effect of the intervention on the outcome is affected by wealth, i.e. is there less of an effect in higher wealth groups and more of an effect in the lower wealth groups.

Neither wealth index influenced the effect of the cash transfer program on HIV or HSV-2 prevalence for either the baseline schoolgirls or baseline dropouts (p>0.05). The interactions between wealth item x intervention and wealth family x intervention were not significant in baseline dropouts for HSV-2 prevalence (p = 0.81 and p = 0.63, respectively) or HIV prevalence (p = 0.44 and p = 0.61 respectively). However, in the baseline schoolgirls, when we looked more closely at the two intervention arms, we found a significant interaction between the wealth family and intervention for the UCT arm for both HIV and HSV-2 prevalence. In a post-hoc analysis, we categorized the wealth family variable into low or high groups (divided at median value of schoolgirls). The odds of HSV-2 was less in the UCT group compared to control in both the wealth family high and low groups, based on point estimates and CIs (Table 4). However, the odds of HSV-2 were even smaller in the UCT group when the wealth family level is low, indicating that the UCT intervention was highly effective when the wealth family is low. Results for HIV are more ambiguous based on the median cutpoint for the wealth family variable.

**Table 4:** Effects of categorized wealth index on HIV and HSV-2 prevalence by intervention arm in baseline schoolgirls.

		Odds Ratio*	95% CI	P Value
Wealth Family (HIV)				
	CCT low^	0.1602	(0.0150, 1.7111)	0.1282
	CCT high	0.1090	(0.0118, 1.0094)	0.0510
	UCT low	0.2005	(0.0365, 1.1027)	0.0644
	UCT high	0.2934	(0.0557, 1.5448)	0.1462
Wealth Family (HSV2)				
	CCT low	0.0723	(0.0048, 1.0949)	0.0580
	CCT high	0.2847	(0.0710, 1.1414)	0.0757
	UCT low	0.0070	(0.0023, 0.0211)	< .0001
	UCT high	0.1335	(0.0165, 1.0776)	0.0586

Note:

*All categories are compared to control group.

^low indicates lower levels of wealth

#### Causal pathway

**Fig 2:**
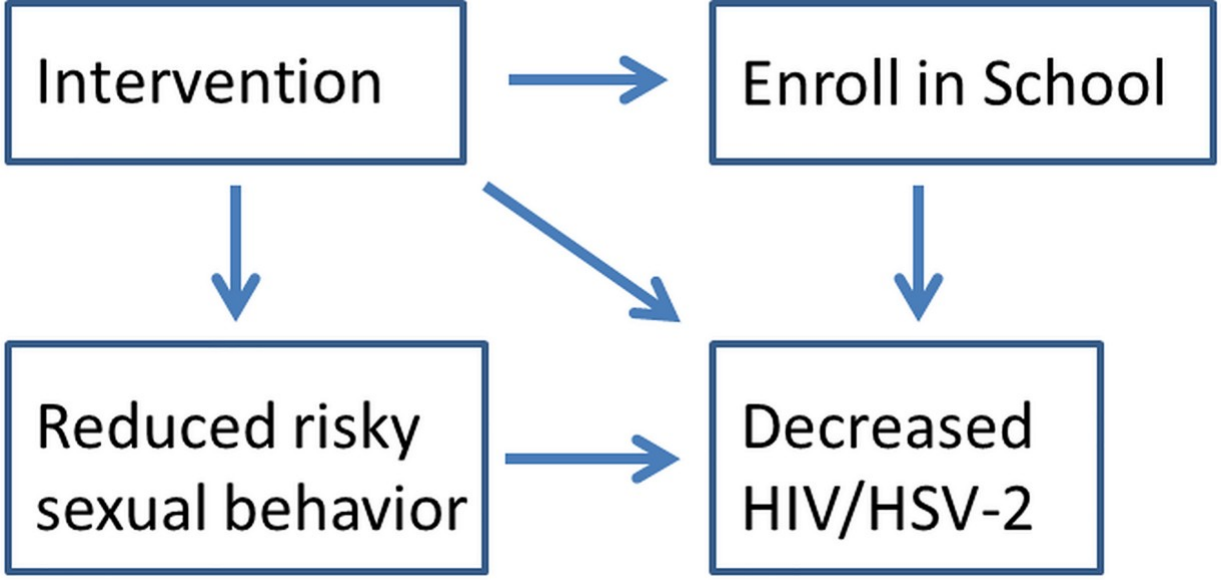
Causal pathway for reduced HIV/HSV-2 prevalence.

The authors of the original paper examined whether the intervention had an effect on whether the participant enrolled in school in 2008, on the prevalence of risky sexual behaviors and on the prevalence of HIV and HSV-2 at 18 months. Here, the direct relationship between school enrollment, risky sexual behaviors (sexual debut, had unprotected sexual intercourse, had sexual intercourse once per week, and had a sexual partner aged ≥25 years) with HIV and HSV-2 prevalence are examined (Fig 2).

The intervention of cash transfers lasted from baseline to 24 months; enrolled in school in 2008 and sexual behaviors are measured at 12 months; and prevalence of HIV and HSV-2 are both measured at 18 months. Since enrolled in school in 2008 and sexual behaviors are measured before HIV and HSV-2, it should be valid to look at the association between these variables. Baird *et*.*al*. have looked extensively at the connection between the intervention and school enrollment, but the direct link between enrollment in school and risky behaviors with HIV/HSV-2 prevalence has not been assessed in this study. Associations between enrolled in school in 2008 and risky behaviors can also be examined, but a cause and effect relationship cannot be established since they were measured at the same time. There are many potential pathways for how the intervention effects HIV and HSV-2 prevalence; however, we pre-specified the pathway shown in Fig 1 and we test only these relationships.

When examining possible mediators, the analysis concentrated on the baseline schoolgirls. Baseline dropouts were excluded from the analysis, since the effects of the cash transfer program on HIV and HSV-2 prevalence were not statistically significant and therefore not subject to mediation. School enrollment, had unprotected sexual intercourse, had sexual intercourse once per week, and had sexual partner aged ≥25 years were considered as possible mediators of the effects of intervention on HIV prevalence or HSV-2 prevalence.

Test 1 show the significant effect of intervention on HIV and HSV-2 prevalence; in baseline schoolgirls, as observed in the original results. Test 2 shows the intervention is significantly associated with school enrollment, had a sexual partner aged ≥25 years, and had sexual intercourse once per week. Intervention was not significantly associated with had unprotected sexual intercourse; therefore, it is no longer considered a mediating variable. Test 3 shows all the potential mediating variables are associated with both HIV and HSV-2 prevalence.

In Test 4, for HIV prevalence, mediation is challenging to assess, because, for both the mediator variable and the intervention, the p-values hover near 0.05, just above or below it. Based on the marginal p-values in this analysis, we conclude that enrolled in school, Had a sexual partner aged ≥25 years, Had sexual intercourse once per week are potential mediators of intervention on HIV prevalence and that more work in this area needs to be conducted (Table 5). Looking at HSV-2, enrollment in school, sexual partner aged ≥25 years, and had sexual intercourse once per week resulted in partial mediation between the effects of intervention and HSV-2 prevalence, indicating that the intervention effect on HSV-2 prevalence is not fully explained by school enrollment or by sexual behaviors (Table 5).

**Table 5:** Mediator analysis of HIV and HSV-2 prevalence.

			Test 1	Test 2	Test 3	Test 4		Conclusion
						Intervention	Mediator	
HIV prevalence								
	Enrolled during 2008 school year	OR (95%CI)	0.4 (0.1–0.9)	1.6 (1.1–2.5)	0.2 (0.04–0.9)	0.4 (0.2–1.1)	0.2 (0.05–1.0)	Potential
		p-value	0.033	0.023	0.041	0.066	0.052	
	Had a sexual partner aged ≥25 years	OR (95%CI)	0.4 (0.1–0.9)	0.2 (0.1–0.6)	7.5 (1.3–43.0)	0.4 (0.2–1.0)	5.9 (1.0–33.9)	Potential
		p-value	0.033	0.005	0.023	0.051	0.048	
	Had unprotected sexual intercourse	OR (95%CI)	0.4 (0.1–0.9)	1.1 (0.7–1.8)	5.9 (2.3–15.0)	-	-	None
		p-value	0.033	0.76	<0.001	-	-	
	Had sexual intercourse once per week	OR (95%CI)	0.4 (0.1–0.9)	0.5 (0.3–0.8)	3.5 (1.2–10.4)	0.4 (0.2–1.0)	3.0 (0.9–9.2)	Potential
		p-value	0.033	0.009	0.027	0.050	0.062	
HSV-2 prevalence								
	Enrolled during 2008 school year	OR (95%CI)	0.2 (0.1–0.7)	1.6 (1.1–2.5)	0.2 (0.1–0.6)	0.3 (0.1–0.8)	0.3 (0.1–0.7)	Partial
		p-value	0.006	0.023	0.004	0.016	0.008	
	Had a sexual partner aged ≥25 years	OR (95%CI)	0.2 (0.1–0.7)	0.2 (0.1–0.6)	9.1 (3.3–25.2)	0.3 (0.1–0.7)	7.2 (2.7–19.1)	Partial
		p-value	0.006	0.005	<0.001	0.009	<0.001	
	Had unprotected sexual intercourse	OR (95%CI)	0.2 (0.1–0.7)	1.1 (0.7–1.8)	6.5 (2.8–14.9)	-	-	None
		p-value	0.006	0.76	<0.001	-	-	
	Had sexual intercourse once per week	OR (95%CI)	0.2 (0.1–0.7)	0.5 (0.3–0.8)	5.1 (1.7–15.5)	0.3 (0.1–0.7)	4.4 (1.4–13.5)	Partial
		p-value	0.006	0.009	0.005	0.009	0.009	

Notes:

Test 1: Intervention effect outcome (HIV or HSV-2 prevalence)

Test 2: Intervention effect on mediator (enrolled in school, sexual behavior)

Test 3: Mediator (enrolled in school, etc.) effect on outcome (HIV or HSV-2 prevalence)

Test 4: Intervention and mediator effect on outcome

## Discussion

The pure replication reproduced the results of the original paper very well, with a few minor discrepancies. There were some typographical errors, some discrepancies in the reported numbers of EAs and group sample sizes, and two point estimates did not match. However, these did not change their significance. The original authors are aware of the typographical errors, but as of December 6, 2018 there has not been a correction published in the Lancet. Despite these discrepancies, the pure replication leads to the same conclusions as the original authors, after accounting for strata and subpopulation/domain. The original findings suggest that the cash transfer program was effective in reducing the prevalence of HIV and HSV-2 in unmarried schoolgirls currently in Malawi. There was no significant reduction of HIV or HSV-2 prevalence for baseline dropouts.

The original authors state that they conducted an intent to treat analysis, but this cannot be verified due to a lack of available protocol and the number of baseline schoolgirls in the intervention group excluded from the analysis. Among the intervention group, a large number of baseline schoolgirls received no cash transfer, based on proportions of 0%, 33%, 66% and 100%. This was done to test the effect of the intervention on untreated schoolgirls in EAs randomized to intervention, essentially the spillover effect. At the 12 month analysis of secondary endpoints, the original authors found no intervention effect among the schoolgirls who did not receive cash transfers, so the elected to not test this group of schoolgirls for HIV and HSV-2 at 18 months. However, since these girls were randomized to the intervention group, for an intent to treat analysis, they should have been included in the primary analysis and received the biological testing. Without an available protocol, it is unclear if this group of schoolgirls was pre-specified to be excluded from an intent-to-treat analysis.

In examining the flow diagram from the original study, another notable problem was discovered. The authors state that 104 EAs were randomly selected for biological testing which would corresponds to 52 intervention and control EAs. We expect that 52 (59%) of the control EAs were selected randomly, but in the intervention group, 27 (100%) out of 27 total UCT EAs were selected and 25 (54%) out of 46 CCT EAs were selected. It is unlikely that this was a random sample of EAs in the intervention group considering 100% of the UCT EAs were selected. Additionally, the baseline dropout cohort had a more balanced randomization scheme (as one would expect) and their results showed virtually no sensitivity to model selection. We therefore question the validity of the sampling scheme in the baseline school girls; especially since the raw data were not available, thereby, limiting our ability to determine how sampling influenced the results.

The MEA portion of the replication study examined valid, alternative methodology for estimation in cluster randomized trials. Alternative methods include GEE, GLMM, and permutation tests. The results from the GEE models tended to agree with the original analysis, which we might expect due to their similarity to the original analysis methods. When examining the sensitivity of model selection with GLMM and permutation testing some differences were identified. The effect of intervention on HIV prevalence in the baseline schoolgirls was not statistically significant in the GLMM analysis (p = 0.25) or by permutation testing (p = 0.07), and confidence intervals and point estimates were different from the original methods. The effect of intervention on HSV-2 prevalence was found to be more robust in terms of statistical significance, though confidence intervals and point estimates were quite different for this outcome as well. Small numbers of HIV and HSV-2 events are likely contributing to these differences, making variance estimates and confidence intervals unstable.

At this point, one might ask, which is the best method? Which analysis should be preferred? A literature search provided no comprehensive comparisons of the competing methods for cluster randomized trials with a binary outcome and sampling weights. Green and Vavreck compare the robust standard errors methodology to GLMM random effects methodology in a simulation study [25]. They found that robust standard errors methodology produces standard errors that are too small when the number of clusters are small, but become less biased as the number of clusters increase. This is compared to standard errors from GLMM which were found to be less biased in this situation. However, Pfeffermann and colleagues recommend caution using GLMM when within cluster sample sizes are small, because variance component parameters can become biased, although scaling the weights reduces that bias[17]. Peters and others compared robust standard errors methodology to GLMM random-effects logistic regression in a sensitivity analysis with actual trial data[26]. Since this was not a simulation, the true parameter estimates are unknown, but we can compare the effects of covariate adjustment on the parameter estimates and standard errors. They found that the parameter estimates were similar between the two methods; however, adjustment for covariates drastically decreased the standard errors in the robust standard errors methodology. In the GLMM random effects models, the effect of covariate adjustment had only a minor effect on the standard error estimates. This indicates that there is not a preferred analysis method for cluster randomized trials.

In Webb and others’ Lancet commentary, the authors state that “the point estimate without clustering had a very wide CI and was not significant and only after significant adjustment was there a significant finding” [19]. In that same Lancet commentary, the original authors reply that “sampling weights are used to account for the fact that younger girls and those living in urban areas were sampled at a lower rate in the study design”[27]. Since the design of the study incorporates multistage sampling and unequal sampling probabilities, the analysis must include those components to have unbiased results. Crude ORs that are not adjusted for the sampling design can be calculated based on the data provided, but will be biased. To address Webb’s criticism we used group permutation-based methods. These methods can be employed when asymptotic theory does not apply, for example with small sample sizes. The real advantage is that they require few distributional assumptions. Although these methods may not be as powerful as parametric methods, there are instances where they have greater power[28]. For HIV prevalence in baseline school girls, the permutation test resulted in an unadjusted p-value of 0.11 and an age geographic stratum adjusted p-value of 0.07, showing marginal associations between intervention and HIV prevalence. For HSV-2 prevalence in baseline schoolgirls the unadjusted p-value is 0.009 and the adjusted p-value of 0.01. The permutation test results for HIV prevalence fall in between the GEE and GLMM results.

Given the large number of clusters randomized in this trial, all the methods accounting for the study design and weighting should give valid and similar results. We found inconsistency in estimation results for HIV prevalence, which could be caused by the extremely low number of baseline schoolgirls with HIV at the end of the study, with only 7 in the intervention group and 17 in the control group. This indicates that there were many clusters with no events. The original paper also points out this finding as a limitation to their study and recommends interpreting the results with caution[8]. This problem was also seen in the HSV-2 outcome, but the effect size was so large for this comparison that the significance did not change based on estimation method. Based on the lack of large-scale simulation studies comparing the various estimation techniques for cluster randomized trials, it is a good idea to compare the results of all the valid techniques and report these results as a sensitivity analysis[29].

Next, we examine the way that the different types of models are interpreted and compared them in that context. GEE analysis is similar to GLMM, except it provides a population average interpretation [30]. This population average approach describes changes in the population mean outcome given a change in the predictor. For example, the intervention effect on HIV in a GEE model gives the odds ratio for intervention compared to control averaging across EA. This differs from a GLMM interpretation, which is known as a conditional model. A GLMM model in our example can be interpreted as odds ratio for HIV prevalence for intervention compared to control in a particular EA (holding the EA of interest constant), lending itself to a more individual interpretation[31]. In choosing a model for policy interventions and populations at as a whole, and a public health standpoint, the GEE model is appropriate. However, it is unclear if the original intent of the study was hypothesis testing at the population level or at the individual level, this lack of clarity makes model choice ambiguous. In the theory of change analyses, the intervention did not have a significant effect on HIV awareness for either baseline schoolgirls or dropouts, and awareness at 12 months was found to be highly associated with baseline level awareness. No interaction was found to exist between the constructed wealth indices and intervention on HIV or HSV-2 in baseline schoolgirls or dropouts. However, when looking at the UCT group, there was a significant interaction between the wealth index for family and the UCT for both HIV and HSV-2 prevalence. UCT had the greatest effect in individuals with low wealth family. However, these results are based on a very small number of events so should be treated with caution.

In the analysis of the causal pathway, school enrollment, had sexual intercourse once per week, and had sexual partner aged ≥25 years were found to be partial mediators of HSV-2 prevalence in baseline schoolgirls. The pathway analysis for HIV prevalence was ambiguous, based on the borderline significant results. Enrolled in school, had a sexual partner aged ≥25 years, and had sexual intercourse once per week are potential mediators of HIV prevalence. This indicates that the effect of cash transfer intervention effect on HIV prevalence is indirect and could be working through its effect on these three variables. Based on these results, we can infer that the intervention is affecting HIV and HSV-2 prevalence at least partially through school enrollment and selected sexual behaviors.

One limitation of this replication is that we only had available the cleaned data set with deidentified individuals from the original authors. Because of this, the original sampling design was used, and it is possible that the results are due to features of the sampling methodology which couldn’t be replicated (such as computing the sampling weights). To overcome this limitation, a clustered permutation test that does not have any distributional assumptions was performed.

## Conclusions

The effect of intervention on HIV prevalence in the baseline schoolgirls was sensitive to the model being used; however, the HSV-2 results were found to be more robust in terms of statistical significance, though the odds ratios and confidence intervals differed. Further, it could not be determined if the main results were influenced by the sampling design. We therefore recommend that the results of the original analysis should be treated with caution, especially for the HIV outcome in baseline schoolgirls, considering the conflicting results based on model choice.
